# Catalysis of the Oxygen-Evolution Reaction in 1.0 M Sulfuric Acid by Manganese Antimonate Films Synthesized via Chemical Vapor Deposition

**DOI:** 10.1021/acsaem.4c00135

**Published:** 2024-03-25

**Authors:** Jacqueline A. Dowling, Zachary P. Ifkovits, Azhar I. Carim, Jake M. Evans, Madeleine C. Swint, Alexandre Z. Ye, Matthias H. Richter, Anna X. Li, Nathan S. Lewis

**Affiliations:** † Division of Chemistry and Chemical Engineering, California Institute of Technology, Pasadena, California 91125, United States; ‡ Division of Chemistry and Chemical Engineering and Beckman Institute, California Institute of Technology, Pasadena, California 91125, United States; § Division of Engineering and Applied Sciences, California Institute of Technology, Pasadena, California 91125, United States

**Keywords:** electrolysis, oxygen-evolution reaction in acid, manganese antimony oxide, earth-abundant materials, heterogeneous catalysis, chemical vapor deposition

## Abstract

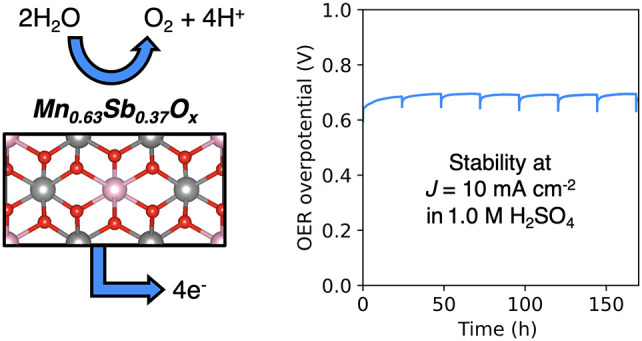

Manganese antimonate (Mn_*y*_Sb_1–*y*_O_*x*_) electrocatalysts for the oxygen-evolution reaction (OER) were synthesized via chemical vapor deposition. Mn-rich rutile Mn_0.63_Sb_0.37_O_*x*_ catalysts on fluorine-doped tin oxide (FTO) supports drove the OER for 168 h (7 days) at 10 mA cm^–2^ with a time-averaged overpotential of 687 ± 9 mV and with >97% Faradaic efficiency. Time-dependent anolyte composition analysis revealed the steady dissolution of Mn and Sb. Extended durability analysis confirmed that Mn-rich Mn_*y*_Sb_1–*y*_O_*x*_ materials are more active but dissolve at a faster rate than previously reported Sb-rich Mn_*y*_Sb_1–*y*_O_*x*_ alloys.

The electrochemical oxygen-evolution reaction (OER) is an anodic process that oxidizes water, an abundant feedstock, and can supply electrons necessary to drive many fuel-forming cathodic processes including the production of H_2_ from H_2_O, NH_3_ from N_2_, and hydrocarbons from CO_2_.^1−3^ Water electrolysis for H_2_ generation specifically is of interest in the storage of energy from intermittent renewable sources.^4,5^ Carbon-free electricity can drive water electrolysis to generate green H_2_ for use on demand.^6,7^ Commercial proton-exchange membrane (PEM) electrolyzers use Ir-based catalysts to effect the oxygen-evolution reaction (OER) in acidic media.^8,9^ IrO_*x*_ exhibits high OER activity and durability, but the low crustal abundance of Ir is a barrier to scale.^10−14^ An earth-abundant, but less-active, electrocatalyst may be an acceptable replacement for IrO_*x*_ in scenarios with infrequent electrolyzer use and low-cost electricity.^10^ Electrolyzers paired with seasonal or multiyear H_2_ storage in reliable wind and solar systems may operate at reduced capacity factors (∼50%) and capitalize on abundant, otherwise-curtailed, zero-cost electricity to drive electrolysis.^5,10,15^

A variety of earth-abundant materials have displayed relatively stable oxygen-evolution catalysis in acidic aqueous electrolytes, including Mn-oxyhalides, arc-melted Ni_2_Ta electrodes, Co-doped Fe_2_O_3_ thin films, and N_2_-doped W-carbide nanoarrays.^16−19^ Earth-abundant Mn-rich rutile Mn_*y*_Sb_1–*y*_O_*x*_ powders are effective catalysts for chemical oxygen-evolution in acidic media, and Sb-rich rutile Mn_*y*_Sb_1–*y*_O_*x*_ sputtered films have shown promising long-term durability.^20,21^ Rutile Mn_*y*_Sb_1–*y*_O_*x*_ (0.3 < *y* < 0.7) materials are more active and stable than nonrutile Mn_*y*_Sb_1–*y*_O_*x*_ materials.^20−25^ In this work, Mn_0.63_Sb_0.37_O_*x*_ was synthesized via chemical vapor deposition (CVD). CVD is a scalable synthetic method and may be an effective approach to controllably coat catalyst layers onto high surface-area supports, including those suitable for use in a PEM electrolyzer.^26^ The CVD deposition method complements previous synthetic routes for generation of Mn_*y*_Sb_1–*y*_O_*x*_ including sputtering, bulk powder mixing, and electrodeposition.^20−23^

Mn_0.63_Sb_0.37_O_*x*_ thin films were deposited by CVD on fluorine-doped tin oxide (FTO) substrates using 30 supercycles that each consisted of 10 SbO_*x*_ subcycles and 5 MnO_*x*_ subcycles (Scheme 1).^27^ Each chemical vapor deposition subcycle consisted of a precursor pulse with either tris(dimethylamido)antimony(III) (TDMA-Sb) or bis(ethylcyclopentadienyl)-manganese (Mn(EtCp)_2_), in addition to an ozone coreactant pulse. The growth rates of MnO_*x*_ and SbO_*x*_ were independently measured via ellipsometry (Figure 1A). The MnO_*x*_ thickness increased linearly with pulse duration, indicating controlled chemical vapor deposition, whereas the thickness of the SbO_*x*_ was constant regardless of the pulse duration, indicating self-limiting atomic-layer deposition.^28^ The Mn–Sb binary oxide was formed using a 0.33 s pulse of Mn(EtCp)_2_, which corresponded to 0.43 nm of MnO_*x*_ per cycle, and a 1 s pulse of TDMA-Sb, which corresponded to 0.12 nm of SbO_*x*_ per cycle. Inductively coupled plasma mass spectrometry (ICP-MS) indicated that the composition of the as-deposited, unannealed catalyst was Mn/(Mn+Sb) = 0.63 ± 0.01. After annealing in air for 6 h at the maximum tolerable temperature (600 °C) of the TEC8 FTO substrate, grazing incidence X-ray diffraction (GIXRD) analysis of Mn_0.64_Sb_0.36_O_*x*_ showed reflections at 2θ ≈ 27°, 35°, 53°, and 56°, consistent with a rutile crystal structure based on a comparison to the reflections of rutile MnSb_2_O_6_.^20,21,23^

**Scheme 1:**
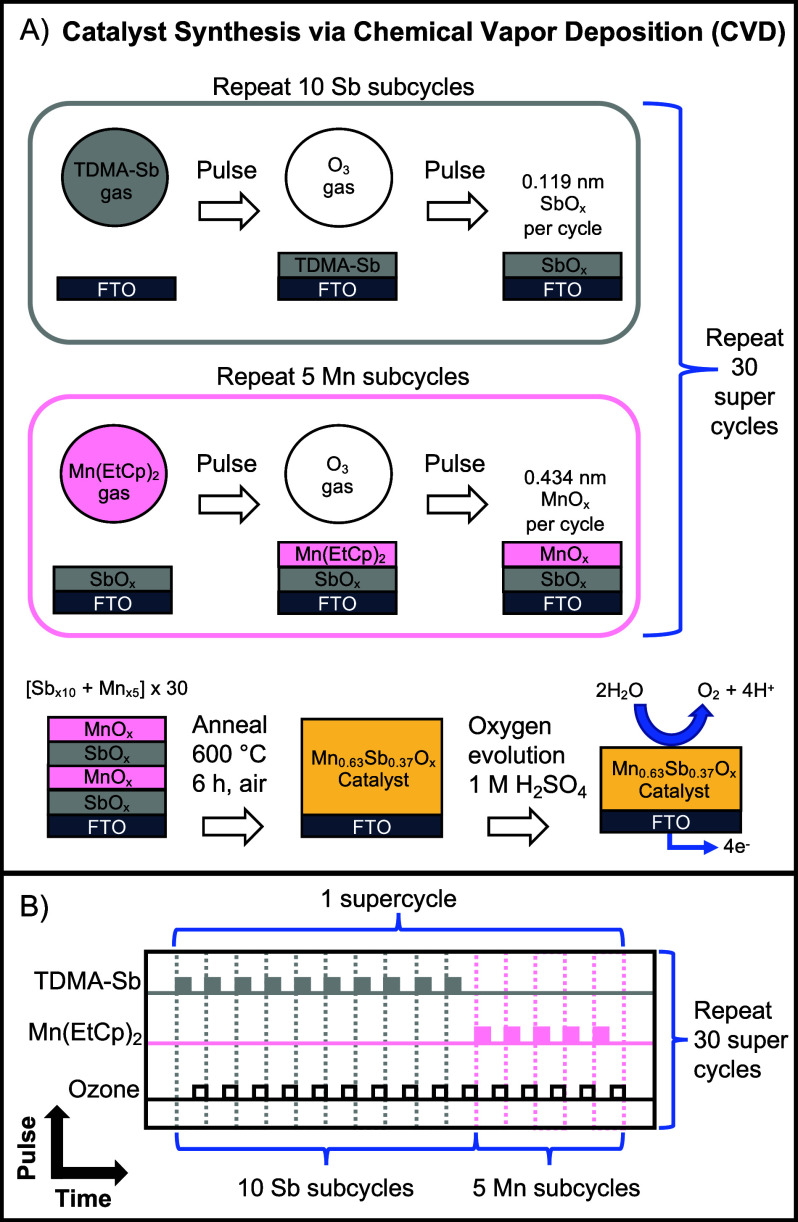
(A) Synthesis of Crystalline Mn_0.63_Sb_0.37_O_*x*_ via Chemical Vapor Deposition and Annealing. (B) Ternary Chemical Vapor Deposition with TDMA-Sb and Mn(EtCp)_2_ Precursors, in Addition to Ozone As a Coreactant

**Figure 1:**
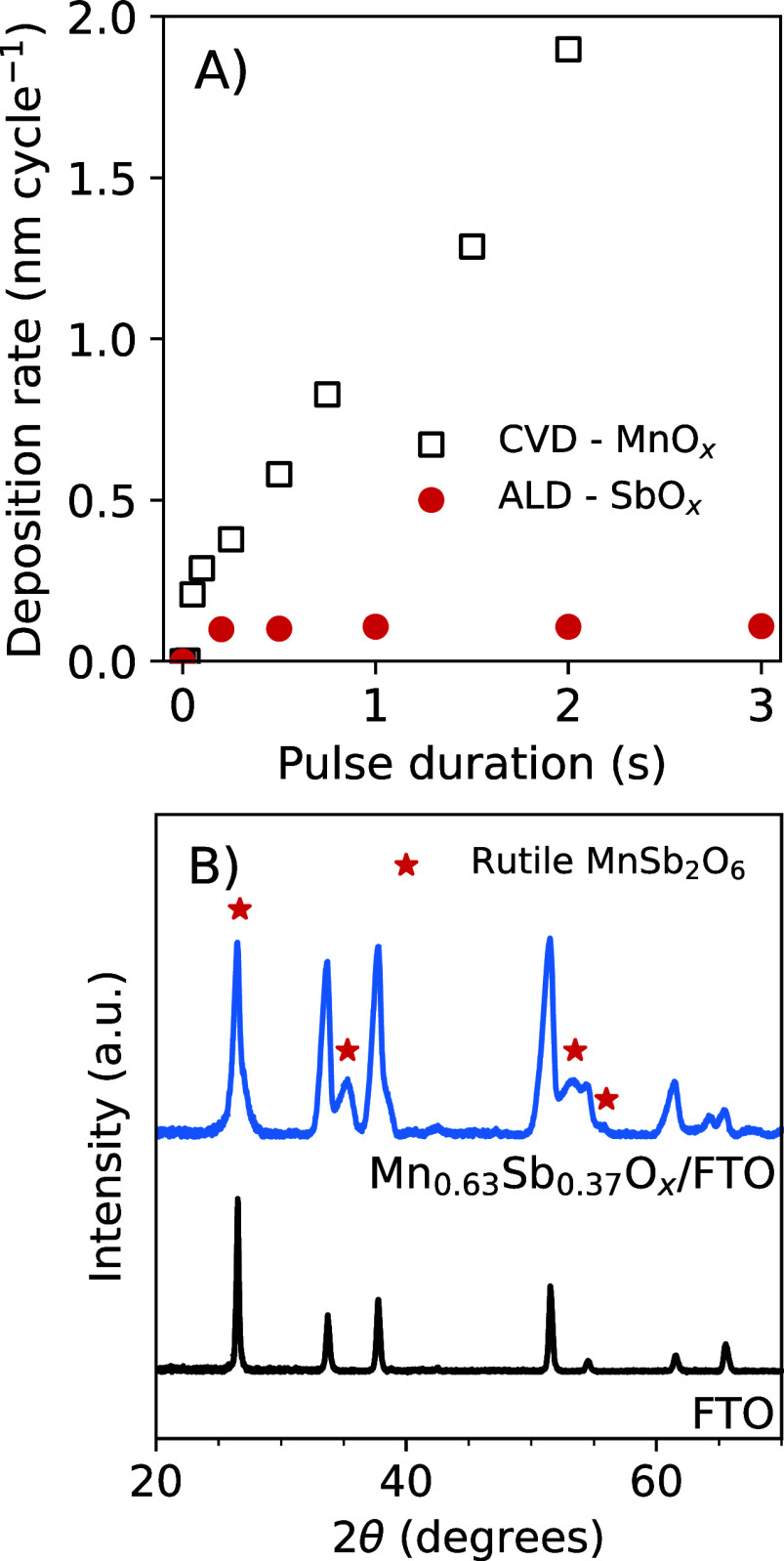
(A) Growth rates of MnO_*x*_ and SbO_*x*_ via CVD and ALD, respectively, derived from the analysis of ellipsometry data. (B) Grazing incidence X-ray diffraction (GIXRD) data acquired from Mn_0.64_Sb_0.36_O_*x*_ and XRD data acquired from the TEC8 FTO substrate after annealing in air for 6 h at 600 °C.

A Mn_0.63_Sb_0.37_O_*x*_ electrode was subjected to a 168 h (7 day) durability test at *J =* 10 mA cm^–2^ in 1.0 M H_2_SO_4_(aq), and the OER overpotential (η) was recorded (Figure 2A). During this experiment, the galvanostatic hold was interrupted at 24 h intervals, and voltammetric and impedance data were collected after 30 s at open circuit (Figure 2B and Figure S2). The measured overpotentials at *J =* 10 mA cm^–2^ were reduced by ∼14 mV to correct for the uncompensated ohmic resistance intrinsic to the electrochemical cell configuration. The time-averaged OER overpotential over the entire test duration was η = 687 ± 9 mV (the blue shaded region in Figure 2A shows the standard deviation). However, consistent with previous results for Mn_*y*_Sb_1–*y*_O_*x*_, during the short periods at open circuit, as well as between the first and second voltametric cycles collected in succession at each 24 h interval, the OER overpotential decreased and the catalyst “recovered” (Figure 2A, Figure S2B).^21,23^ The OER overpotential at 10 mA cm^–2^ as measured from the voltammetric analyses was η = 617 mV at *t =* 0 h and was η = 618 mV at *t =* 168 h (Figure 2B, Figure S2B). Redox waves centered at 1.46 V vs the reversible hydrogen electrode (RHE) appeared and increased in magnitude, during the extended durability test (Figure 2B, Figure S2A), analogous to the behavior of Mn_*y*_Sb_1–*y*_O_*x*_ electrocatalysts deposited by sputtering.^21^

**Figure 2:**
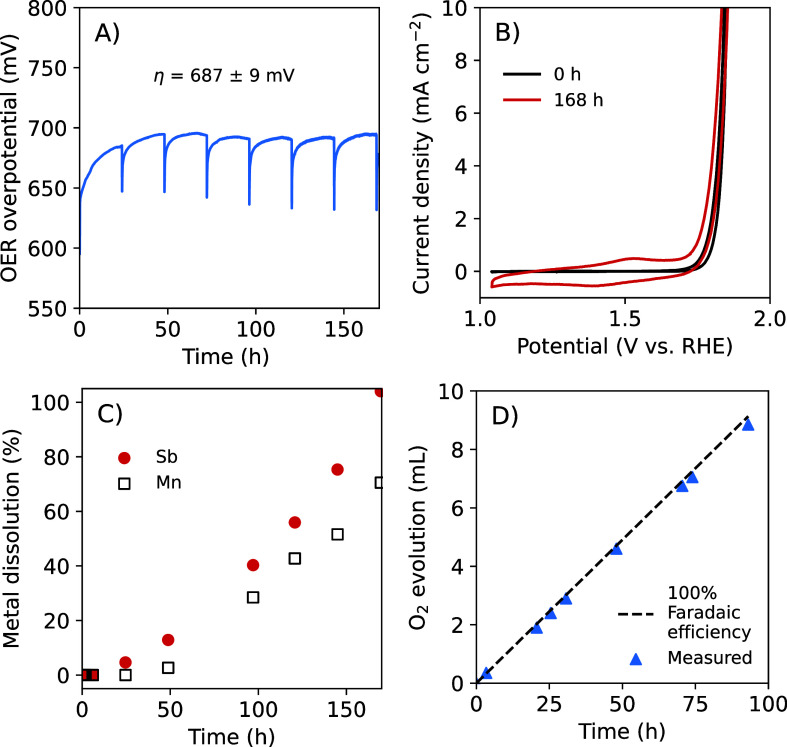
Electrochemical activity, stability, and Faradaic efficiency of Mn_0.63_Sb_0.37_O_*x*_ during the OER at *J* = 10 mA cm^–2^ for 168 h (7 days) in 1.0 M H_2_SO_4_(aq). (A) Time dependence of the OER overpotential after correction for the uncompensated resistance of the cell. (B) Cyclic voltammograms (*v* = 40 mV s^–1^) collected after *t* = 0 h and after *t* = 168 h of the galvanostatic hold. (C) Amount of dissolved metal in the anolyte as quantified by ICP-MS, as a percentage of the total deposited Sb and total deposited Mn. (D) Eudiometric measurement of the level of O_2_(g) production.

Aliquots of the electrolyte solution were taken without replacement at ∼24 h intervals, and the dissolution of Sb and Mn was measured by ICP-MS during the durability test at 10 mA cm^–2^ in 1.0 M H_2_SO_4_(aq) (Figure 2C). The average rate of Sb dissolution (11 weight % per day, or 0.0013 μmol cm^–2^ h^–1^) was comparable to the average rate of Mn dissolution (8% per day, or 0.0015 μmol cm^–2^ h^–1^) (Figure S3). The dissolution rate of both metals was lower during the initial 48 h of the test than at later time points. Another Mn_0.63_Sb_0.37_O_*x*_ electrode from the same deposition batch yielded an average of 97.6% Faradaic efficiency for oxygen evolution during 93 h of continuous operation at 10 mA cm^–2^ in 1.0 M H_2_SO_4_ (Figure 2D). Hence, despite the high Faradaic efficiency and a relatively stable OER overpotential, substantial catalyst corrosion occurred, consistent with the behavior of sputtered Mn-rich alloys.^23,24^ The OER overpotential and metal dissolution rates of a replicate electrode that was tested for 176 h (>7 days) at 10 mA cm^–2^ in 1.0 M H_2_SO_4_ were in agreement with that of the Mn_0.63_Sb_0.37_O_*x*_ electrode described above (Figure S3).

An additional Mn_0.63_Sb_0.37_O_*x*_ electrode was operated galvanostatically at *J* = 100 mA cm^–2^ and was subjected to very positive potentials during voltametric analysis (Figure 3). The time-averaged OER overpotential over a period of 8.5 h at *J* = 100 mA cm^–2^ was 724 ± 8 mV (Figure 3). Figure S8 presents an expanded view of the data in Figure 3 during the first 8 h of operation. The overpotential of the OER at *J* = 100 mA cm^–2^ was 709 mV at *t* = 0 h and 688 mV at *t* = 8 h (Figure 3). In the first 8 h at *J* = 100 mA cm^–2^ in 1.0 M H_2_SO_4_, ICP-MS indicated more leaching of Sb than of Mn (Figure 3C). The chronopotentiometry experiment at 100 mA cm^–2^ in 1.0 M H_2_SO_4_ was continued for 26 h, with periodic interruptions due to bubble formation that inhibited current flow at the counter electrode (Figure 3). Voltammetric analysis indicated that the initial OER overpotential at *J* = 350 mA cm^–2^ was 819 mV in 1.0 M H_2_SO_4_ (Figure S9).

**Figure 3:**
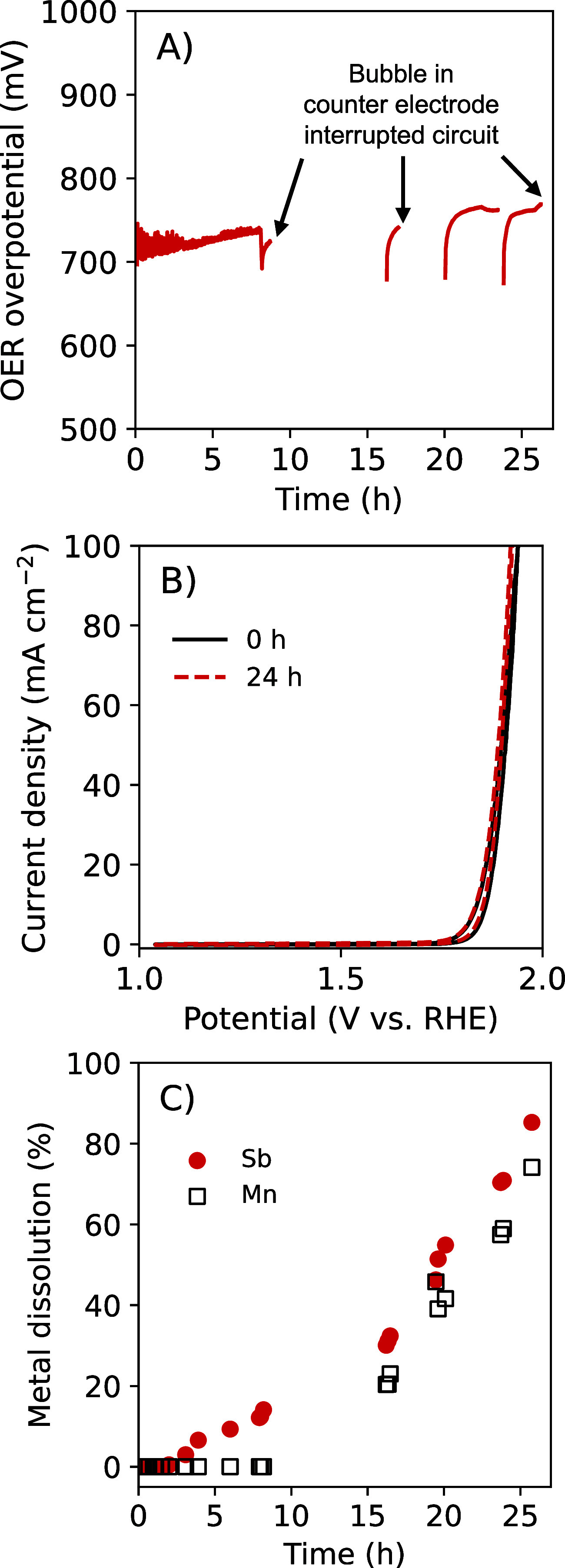
Electrochemical activity and stability of Mn_0.63_Sb_0.37_O_*x*_ during OER at *J* = 100 mA cm^–2^ in 1.0 M H_2_SO_4_(aq). (A) Chronopotentiometric response. (B) Cyclic voltammograms collected initially as well as after 24 h under the galvanostatic hold. (C) Amount of dissolved metal in the anolyte as quantified by ICP-MS, presented as a percentage of the total deposited Sb and total deposited Mn.

The Mn_0.63_Sb_0.37_O_*x*_ electrode was characterized before and after the 168 h of the OER durability test at *J =* 10 mA cm^–2^ in 1.0 M H_2_SO_4_(aq) by scanning electron microscopy (SEM), electrochemical impedance spectroscopy (EIS), energy dispersive X-ray spectroscopy (EDX), and X-ray photoelectron spectroscopy (XPS). The SEM data showed a conformal coating of the catalyst on the substrate prior to the OER, and EIS measurements (Figure S4B and C, respectively) indicated an ∼22-fold increase in surface roughness during the 168 h durability test (Figure S5). Notably, although the material dissolved, the overpotential required to produce *J* = 10 mA cm^–2^ did not change substantially during this time.

The redox waves observed at ∼1.46 V vs RHE (Figure 2B) in the voltammetric data are consistent with behavior of MnO_*x*_ and other Mn_*y*_Sb_1–*y*_O_*x*_ materials.^21,29^ XP spectra of the Mn_0.63_Sb_0.37_O_*x*_ catalyst material acquired before and after the 168 h OER durability test at *J =* 10 mA cm^–2^ in 1.0 M H_2_SO_4_(aq) indicated that the material was always principally composed of Mn(III) with some Mn(IV) observable (∼20%) after operation, consistent with previous analysis of antimonate systems (Figure S6, Figure S7, Table S3).^23^ Sb sites in binary oxide materials are inactive for the OER reaction,^21,23,24^ consistent with the OER being localized on Mn sites and mediated by Mn redox events, with Sb^5+^ ions contributing to electrochemical stability.^21−23^ Electrocatalytically inactive Sb^5+^ sites may stabilize Mn sites that actively effect the OER by inducing enhanced hybridization of the O p-orbital and Mn d-orbital.^23,24^ The Mn metal fraction as indicated by energy-dispersive X-ray (EDX) spectroscopy decreased from 64 ± 5% before operation to 49 ± 7% after 168 h at *J* = 10 mA cm^–2^ (Figure S4A). XP spectra of the Sb 3d region indicated a shift from 3.2 to 5.0 in the Sb oxidation state (Figure S6, Figure S7C, and Table S3). Mn-rich alloys are thus expected to be less stable than Sb-rich alloys, consistent with the substantial metal dissolution of the Mn_0.63_Sb_0.37_O_*x*_ catalysts observed during the multiday durability test (Figure 2). However, some degree of electronic stabilization of Mn sites by Sb ions may account for the enhanced corrosion resistance observed herein relative to that reported for unary Mn oxide materials.^23,24^

In summary, the extended durability of rutile Mn_0.63_Sb_0.37_O_*x*_ catalysts was assessed during galvanostatic operation at *J* = 10 mA cm^–2^ and at *J* = 100 mA cm^–2^ in 1.0 M H_2_SO_4_. After 168 h of operation at *J* = 10 mA cm^–2^, a loss of electrocatalyst mass, an increase in porosity, and partial oxidation of the constituent Mn were observed relative to the as-prepared material. A lower overpotential was observed for the Mn-rich alloy at *J =* 10 mA cm^–2^ than previously reported for Sb-rich Mn_*y*_Sb_1–*y*_O_*x*_ alloys.^21^ However, unlike the Sb-rich Mn_*y*_Sb_1–*y*_O_*x*_ alloys, Mn_0.63_Sb_0.37_O_*x*_ catalysts corroded continuously during operation. This behavior is consistent with the notion that Sb stabilizes Mn sites, as well as with prior results on the behavior of Mn-rich alloys prepared by sputtering.^23,24^ The extended duration testing reported here, along with previous reports, confirm an activity-stability trade-off across the Mn:Sb composition space.^30^ A reduced Mn:Sb ratio may thus enhance the stability of Mn_*y*_Sb_1–*y*_O_*x*_ catalysts in acidic OER conditions while, however, producing a reduction in the OER activity. Despite the continuous corrosion of both Sb and Mn from the as-prepared material, the OER overpotential at *J* = 10 mA cm^–2^ did not substantially increase, even at the point that >90% of the catalyst mass had dissolved.

## Supporting Information Available

Detailed experimental procedures, materials and chemicals, sample preparation, electrochemical measurements, materials characterization, additional OER overpotential data and metal dissolution rates, scanning electron micrographs, energy-dispersive X-ray spectroscopy data, X-ray diffraction data, and X-ray photoelectron spectra (PDF)
