# Supplementary material for: Catalysis of the Oxygen-Evolution Reaction in 1.0 M Sulfuric Acid by Manganese Antimonate Films Synthesized via Chemical Vapor Deposition
Source: ACS Appl Energy Mater. 2024 Mar 25;7(10):4288–93. doi: 10.1021/acsaem.4c00135 (PMC11134315; doi:10.1021/acsaem.4c00135)
Supplement: Supplementary file 1 — ae4c00135_si_001.pdf [file ae4c00135_si_001.pdf]

Supporting Information for:

Catalysis of the oxygen-evolution reaction in 1.0 M sulfuric acid by manganese antimonate films synthesized via chemical vapor deposition

Jacqueline A. Dowling[†,⊥], Zachary P. Ifkovits[†,⊥], Azhar I. Carim[†,‡], Jake M. Evans[†], Madeleine C. Swint[†], Alexander Z. Ye[†], Matthias H. Richter[†], Anna X. Li[†], and Nathan S. Lewis[†,‡,*]

[†]Division of Chemistry and Chemical Engineering, California Institute of Technology, Pasadena, California 91125, United States

[‡]Beckman Institute, California Institute of Technology, Pasadena, California 91125, United States

[⊥]J. A. D. and Z. P. I. contributed equally.

*Email: nslewis@caltech.edu

Experimental Methods

Materials and Chemicals

The chemical vapor deposition (CVD) precursor, bis(ethylcyclopentadienyl)manganese (98%-Mn, STREM) ($Mn(EtCp)_2$), was preloaded in a CVD cylinder and used as received. The atomic layer deposition (ALD) precursor tris(dimethylamino)antimony (99.99%-Sb, STREM) (TDMA-Sb) was loaded into a new ALD cylinder in a glovebox under N_2(g). TEC 8 fluorine-doped tin oxide (FTO, Sigma Aldrich) substrates were used as received. The electrolyte, H_2SO_4 (95.0-98.0%, ACS reagent grade, JT Baker) was diluted to 1.0 M using 18.2 MΩ cm resistivity water obtained from a Thermo Scientific Nanopure system. In-Ga eutectic (99.99%, metals basis, Alfa Aesar), PELCO conductive Ag paint (Ted Pella, Inc), and Loctite epoxy (EA 9460) were used as received. O_2(g) (ultra-high purity grade, Airgas) was used to produce O_3(g), which was used as a co-reactant in chemical vapor deposition. O_2(g) (industrial grade, Airgas) was used to purge the electrolyte solution during durability experiments. 12.1 M HCl and 15.6 M HNO_3 (ACS reagent grade, Sigma Aldrich) were used to clean glassware.

Sample preparation

Chemical vapor deposition was performed using a Cambridge Nanotech Savannah S200 Atomic Layer Deposition (ALD) System. After the precursors and co-reactants were pulsed, a waiting period allowed the ALD chamber (set at 150 °C) to return to the base vacuum pressure (~0.5 torr) at a 20 sccm N_2 flow rate. Precursor cylinder jackets were heated as noted (Table S1).

After consecutive rinsing with isopropyl alcohol, acetone, and H_2O, and drying with N_2(g), the TEC8 fluorine-doped tin oxide (FTO) substrates were loaded into the ALD chamber. Prior to deposition, a glass slide was positioned in the ALD chamber on top of the FTO substrate. The slide covered part of the surface, allowing for subsequent top-facing electrical contact directly to the substrate during the fabrication of electrodes (Figure S1). To anneal the samples, the temperature of a Thermolyne muffle furnace was increased from room temperature to 600 °C at a rate of 10 °C min^{-1}, and then was held for 6 h at 600 °C before being allowed to return to room temperature.

CVD subcycle	
Precursor	Co-reactant
Pulse time	Pulse time
Wait time	Wait time
Jacket temp	Jacket temp
Number of subcycles	

(

SbO_x subcycle	
TDMA-Sb	O_3
1 sec	0.09 sec
25 sec	25 sec
55 °C	25 °C
Repeat for 10 subcycles	

+

MnO_x subcycle	
Mn(EtCp$_2$)	O_3
0.33 sec	0.06 sec
15 sec	15 sec
100 °C	25 °C
Repeat for 5 subcycles	

)

Repeat for 30 supercycles

Table S1. Chemical vapor deposition recipe for $Mn_{0.63}Sb_{0.37}O_x$.

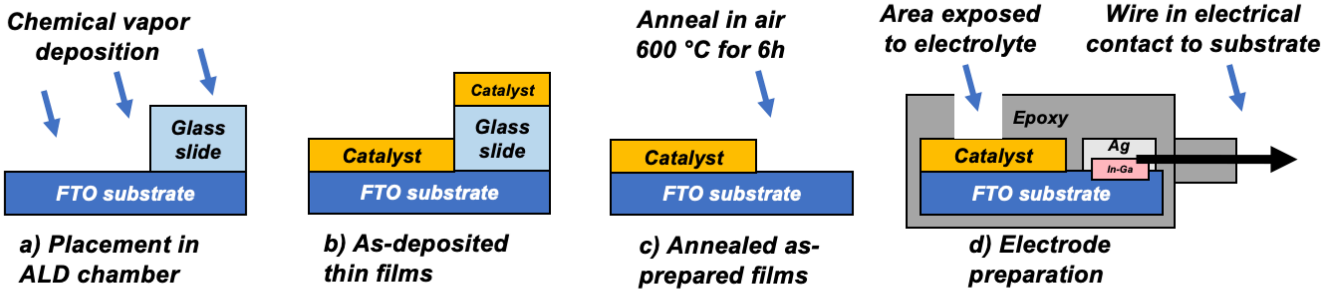

Figure S1. Electrode preparation schematic. A glass slide partially covered the FTO substrate to allow for subsequent electrical contact directly to the substrate.

Annealed $Mn_{0.63}Sb_{0.37}O_x$ samples were cleaved, and In-Ga was scribed directly onto the bare FTO substrate that had been covered in the ALD chamber by a glass slide (Figure S1a). The sample was affixed to tinned Cu wire using Pelco conductive Ag paint (Ted Pella, Inc.), and the assembly was then dried in an oven for 1 h at 95 °C. Except for a small area where the catalyst was exposed, the entire electrode was encased in epoxy, and the entire electrode was dried in an oven for ~ 8 h at 95 °C. The exposed catalyst area (Figure S1d) was measured with an optical scanner (Epson perfection V360), and the geometric area (0.10 - 0.15 cm^2) was quantified using ImageJ software.

Electrochemical measurements

Electrochemical analysis was performed using a Biologic SP-200 potentiostat with EC-Lab software. Experiments were performed in a two-compartment, three-electrode configuration in a 50 mL pyrex flask. The potential of the SCE reference electrode (CHI150, CH Instruments) relative to a reversible hydrogen electrode (RHE) was measured by monitoring the voltage difference between the SCE and an unannealed Pt mesh electrode in 1.0 M H_2SO_4(aq) that was exposed to a continuous stream of H_2(g). The Ti-mesh counter electrode was isolated from the working and reference electrodes using a porous glass frit (porosity of 10-20 μm, Ace glass). All glassware, as well as the Pt mesh, were cleaned by immersion for several hours in a freshly prepared 3:1 (by volume) solution of 12.1 M HCl(aq) and 15.6 M HNO_3(aq), followed by rinsing with H_2O before use for electrochemistry. During all experiments, the 1.0 M H_2SO_4(aq) electrolyte solution (50 mL) was continuously stirred and maintained at room temperature. Water-saturated O_2(g) was continuously bubbled into the solution during electrochemistry.

The electrochemical durability experiment was performed under galvanostatic conditions (J = 10 mA cm^{-2}) for seven 24 h intervals (168 h total). All electrochemical data are presented

using the IUPAC convention. At 24 h intervals during durability tests at 10 mA cm^{-2}, three voltammetric cycles were recorded at a scan rate of v = 40 mV s^{-1}, with an initial potential, E_0 = 1.04 V vs. RHE and an upper-limit potential of E_1 = 1.94 V vs. RHE. Impedance data were collected at open circuit (~1.4 V vs RHE) by applying a 10 mV amplitude sinusoidal wave and measuring 10 data points per decade at frequencies that ranged from 10 Hz to 10 kHz. Impedance data were fit to a modified Randles circuit ($R_1 + Q_2 / R_2$) with EC-lab software to determine the solution resistance (R_1), the capacitance of a constant-phase element (a "leaky" capacitor, Q_2), and the charge-transfer resistance associated with the OER kinetics (R_2). The uncompensated resistance correction was set to equal 90% of the solution resistance (10-15 Ω) measured by impedance. The value of Q_2 was used to evaluate the electrochemically active surface area (ECSA) as well as the change in surface roughness during the durability experiment. For ECSA calculations, the roughness factor (*RF*) of the TEC 8 FTO substrate was assumed to be equal to that of antimony-doped tin oxide (ATO) (*RF* = 1.32).[1] The geometric area-normalized capacitance of ATO (0.0254 mF cm^{-2}) was divided by the roughness factor to determine the capacitance normalized to the electrochemical surface area (0.0192 mF cm^{-2}).[1]

0.2 mL aliquots of electrolyte were taken during galvanostatic operation and were diluted by 5 mL of 5% w/w HNO_3(aq) (0.8 M) before analysis by inductively coupled plasma mass spectrometry (ICP-MS) using an Agilent 8800 Triple Quadrupole ICP-MS system. Standards of known concentration were produced from 10 µg mL^{-1} Mn and 999 ± 2 µg mL^{-1} Sb standards (Sigma Aldrich) via serial dilution with 5% w/w HNO_3(aq) (0.8 M).

Eudiometric measurements indicated > 97% Faradaic efficiency for O_2 production for a $Mn_{0.63}Sb_{0.37}O_x$ electrode during 93 h of continuous operation at J = 10 mA cm^{-2}.

Materials characterization

Ellipsometric analysis was used to measure the growth rates of individual oxides on Si substrates. Data were collected using 65, 70, and 70 ° angles across a wavelength range of 380 – 890 nm with a J.A. Woolam Co. ellipsometer. Ellipsometry data were analyzed using the CompleteEASE software package.

The stoichiometry of the catalyst material was determined by dissolving deposited unannealed metal films on Si substrates for several days in 10 mL of 1.0 M H_2SO_4(aq). The concentration of dissolved metals (Sb and Mn) was then determined using ICP-MS. Films from the same deposition batch were annealed, assembled into electrodes (Figure S1), and subjected to galvanostatic testing (Figure 2, Figure 3). Samples of the anolyte were taken periodically during galvanostatic testing and assessed via ICP-MS to quantify the amount of material that had dissolved into the electrolyte. The values from this ICP-MS analysis were normalized by the concentrations measured when the annealed metal films were fully dissolved, as described above, to yield the percentages of metals in the film that had dissolved at any point in time.

The geometric area of the Si substrates was measured with an optical scanner (using the same procedure used to define the electrode area), to determine the area-normalized mass-loading (23.53 μg Mn cm^{-2}, 32.30 μg Sb cm^{-2}). A mass loading of 0.42 μmol Mn cm^{-2} corresponded to an ~100 nm thick oxide (Table S2).

**Metal**	**Metal fraction (%)**	**Mass loading (μg cm^{-2})**	**Mass loading (μmol cm^{-2})**	**Oxide thickness (nm)**
**Sb**	63%	32.30	0.27	35.7
**Mn**	37%	23.53	0.43	65.1
**Total metal**	100%	55.83	0.70	~100

Table S2. Mass loading and metal fraction of as-prepared $Mn_{0.63}Sb_{0.37}O_x$ catalysts determined by ICP-MS analysis of unannealed films digested in 1.0 M H_2SO_4(aq) for several days. The oxide thickness was based on individual oxide growth rates determined from ellipsometry and the total subcycles used during film growth (Scheme 1).

Scanning-electron micrographs (SEMs) were obtained with a FEI Nova NanoSEM 450 at an accelerating voltage of 10.00 kV with a working distance of 5 mm and an in-lens secondary electron detector. Micrographs were acquired with a resolution of 688 pixels μm^{-1} over ~ 2 μm^2 areas. Energy dispersive X-ray (EDX) spectroscopy was performed in the SEM using an accelerating voltage of 15.00 kV and a working distance of 5 mm, with an Oxford Instruments X-Max silicon drift detector. Spectra were collected in the range of 0 to 10 keV, and quantitative compositions of the deposited material were obtained using the "INCA" software package (Oxford Instruments). Compositions were reported for the average of n = 6 independent measurements from different locations on the sample.

X-ray diffraction (XRD) data were collected using a Bruker D8 Discover diffractometer with a Cu Kα source and a two-dimensional Vantec detector. XRD data were acquired from the FTO substrate in a Bragg-Brentano geometry. Grazing incidence X-ray diffraction (GIXRD) data were collected from $Mn_ySb_{1-y}O_x$ on a FTO substrate with the X-rays directed at a grazing angle ω = 0.3° above the plane of the sample surface, with the detector swept throughout the entire 2θ range.

X-ray photoelectron spectroscopy (XPS) was performed using a Kratos Axis Ultra system with a base pressure of 3×10^{-9} Torr in the analysis chamber. A 150 W monochromatic Al Kα source was used to irradiate the sample with X-rays (1486.6 eV). For maximum depth sensitivity, the hemispherical analyzer was oriented for detection along the sample surface normal. The data were analyzed using CasaXPS software. A Shirley background was used for Mn spectra and a U 2 Tougaard background was used to more accurately capture the background signal for Sb, O, and C spectra. All peaks were referenced to adventitious C at a binding energy of 284.8 eV. The Mn $2p_{3/2}$ peak was fit to standards of pure oxides, to estimate the Mn oxidation state and the

contribution of multiple oxidation states to the peak.[2] Mn 3p spectra were used in conjunction with the Mn $2p_{3/2}$ emissions to more accurately determine the Mn oxidation state.[2–6] The Sb $3d_{5/2}$ peak overlapped the O 1s emission, so the Sb oxidation state was determined from the Sb $3d_{3/2}$ peak, which has no overlap with O. The Sb $3d_{3/2}$ peak was fit with literature standards for the Sb oxidation state, and the Sb $3d_{5/2}$ contribution was calculated by constraining the spin-orbit peak splitting (ΔSb 3d = 9.38 eV), taking the full width at half maximum (FWHM) to be equivalent between the peaks, constraining the peak area ratio to be $3d_{5/2}$:$3d_{3/2}$ = (3:2), and assuming that the remaining signal was due to O 1s.

Additional Data

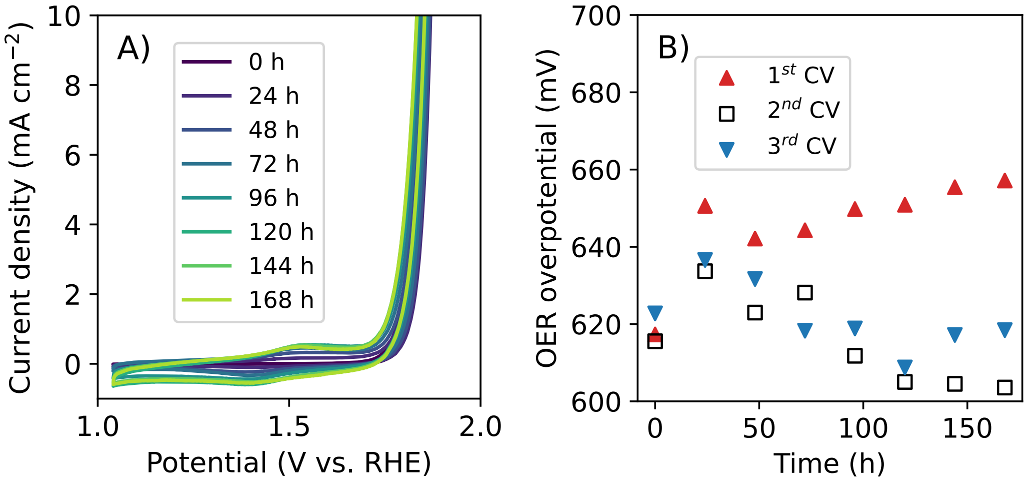

Figure S2. (A) Cyclic voltammograms acquired with a $Mn_{0.63}Sb_{0.37}O_x$ electrode in 1.0 M H_2SO_4(aq) at 24 h intervals by interrupting a continuous galvanostatic hold of $Mn_{0.63}Sb_{0.37}O_x$ at J = 10 mA cm^{-2}. (B) OER overpotential to obtain J = 10 mA cm^{-2} derived from the cyclic voltammetric data in (A).

The IR-compensation was ~ 14 mV. Larger overpotentials were observed in the first voltammogram acquired in each interval relative to the second and third voltammograms, and this effect increased at later time intervals of the experiment (Figure S2B).

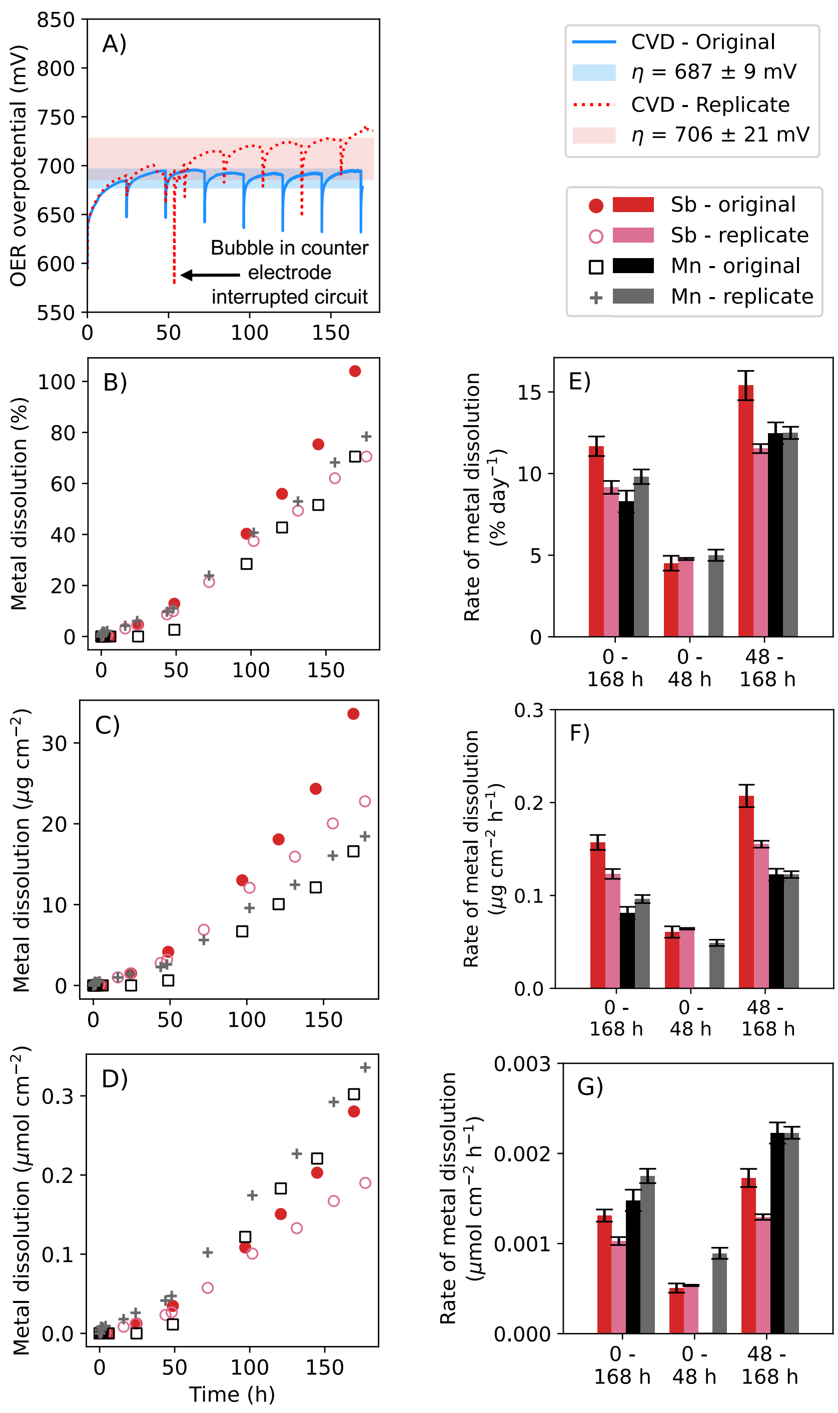

Figure S3. Replicate of $Mn_{0.63}Sb_{0.37}O_x$ dissolution measurements during an OER durability test. (A) The time-averaged OER overpotential was η = 706 ± 21 mV at J = 10 mA cm^{-2} over 176 h in 1.0 M H_2SO_4(aq). ICP-MS measurements of the corrosion products in the electrolyte during the analogous 176 h durability test in relative (B) and absolute terms (C, D). The corresponding bar graphs in panels (E-G) compare metal dissolution rates in various regimes: 0 - 168 h, 0 - 48 h, and 48 - 168 h. Error bars represent the standard error of the dissolution rate given by linear regression over the specified time regime.

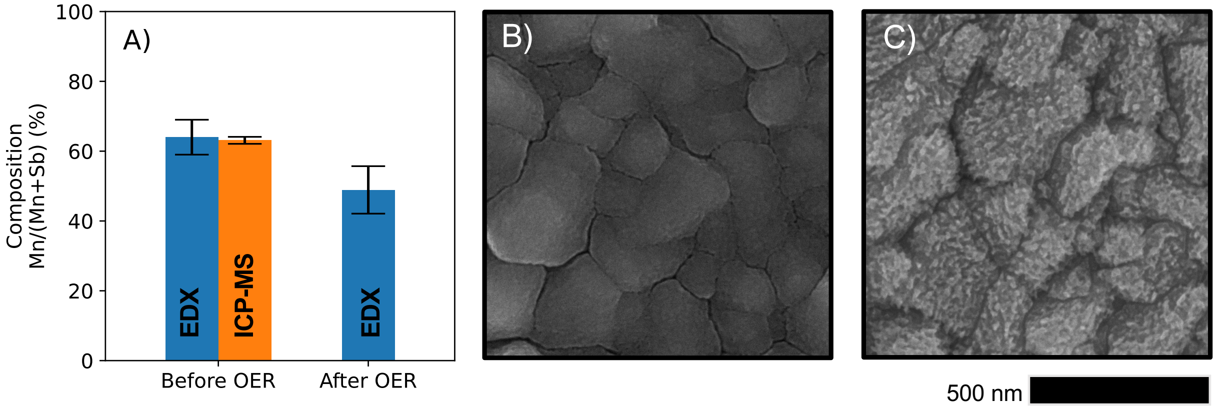

Figure S4. (A) Catalyst composition as determined by EDX and ICP-MS. Representative SEMs of $Mn_{0.63}Sb_{0.37}O_x$ (B) before and (C) after OER at $J = 10$ mA cm^{-2} for 168 h in 1.0 M H_2SO_4(aq). Catalyst composition as determined by EDX after OER was $Mn_{0.49}Sb_{0.51}O_x$.

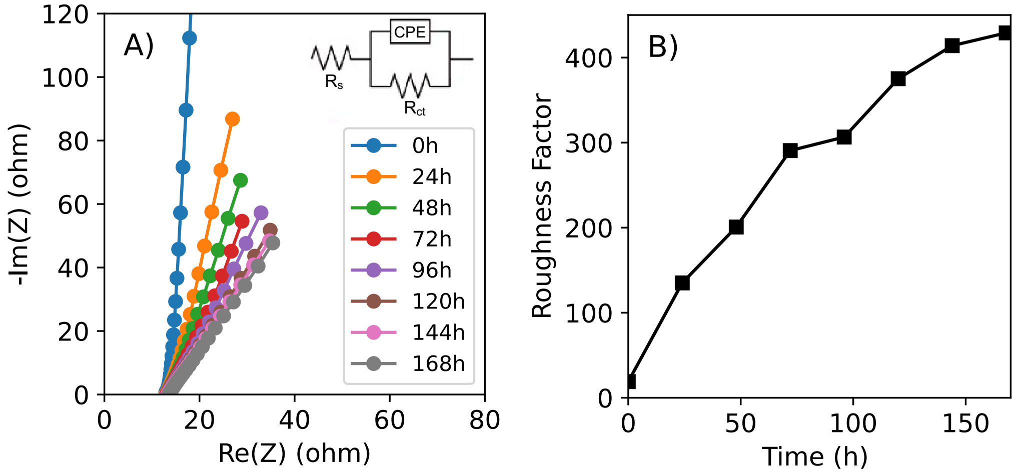

Figure S5. (A) Impedance data and (B) roughness factor for $Mn_{0.63}Sb_{0.37}O_x$ taken at 24 h intervals during OER at $J = 10$ mA cm^{-2} for 168 h in 1.0 M H_2SO_4(aq).

The TEC 8 FTO substrate roughness factor was assumed to be equal to that of ATO (*RF* = 1.32).[1] According to preestablished methods, the geometric area-normalized capacitance of ATO (0.0254 mF cm^{-2}) was divided by the roughness factor to determine the capacitance normalized to the electrochemical surface area (0.0192 mF cm^{-2}).[1]

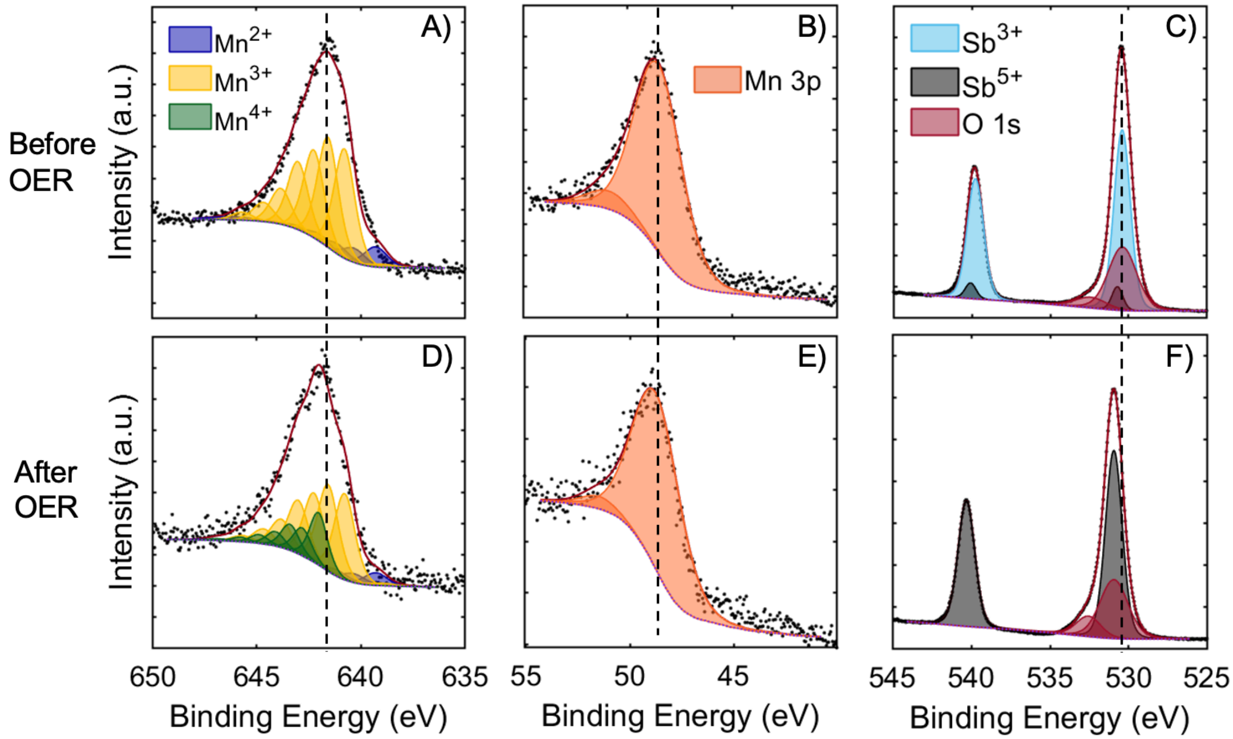

Figure S6. XP spectra of $Mn_{0.63}Sb_{0.37}O_x$ (A-C) before and (D-F) after OER at $J = 10$ mA cm^{-2} for 168 h in 1.0 M H_2SO_4(aq). (A, D) Mn 2p spectra; (B, E) Mn 3p spectra; (C, F) Sb 3d, O 1s spectra. Raw data (black dots) were fit by peak models as described in experimental methods. The sum of all fit peaks (envelope) is denoted by a red line in all panels.

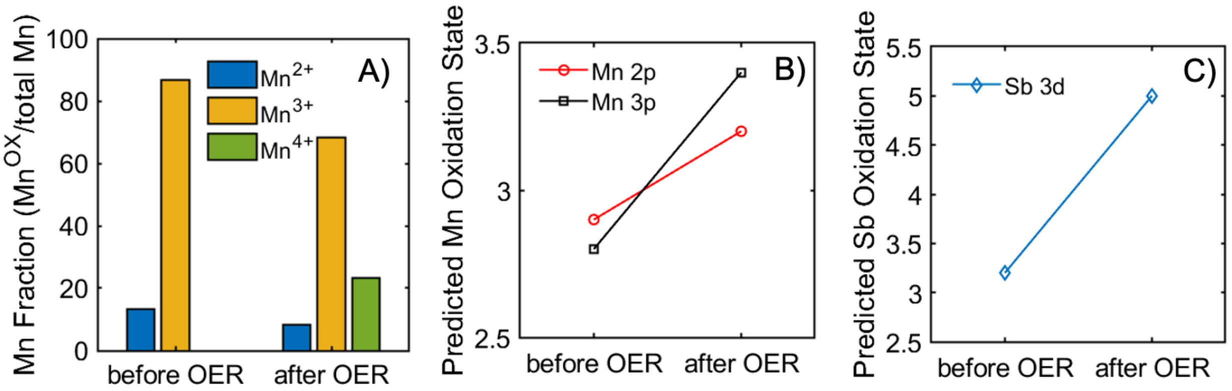

Figure S7. Oxidation state derived from analysis of XP spectra of $Mn_{0.63}Sb_{0.37}O_x$ before and after OER at J = 10 mA cm^{-2} for 168 h in 1.0 M H_2SO_4. (A) Mn oxidation states; (B) derived Mn oxidation states from the 3p and 2p peaks. (C) Sb oxidation states derived from the 3d peak.

	Mn 2p	Mn 3p	Sb 3d
Before OER	2.9	2.8	3.2
After OER	3.2	3.4	5.0

Table S3. Oxidation state of $Mn_{0.63}Sb_{0.37}O_x$ derived from analysis of XP spectra before and after OER at J = 10 mA cm^{-2} for 168 h in 1.0 M H_2SO_4(aq).

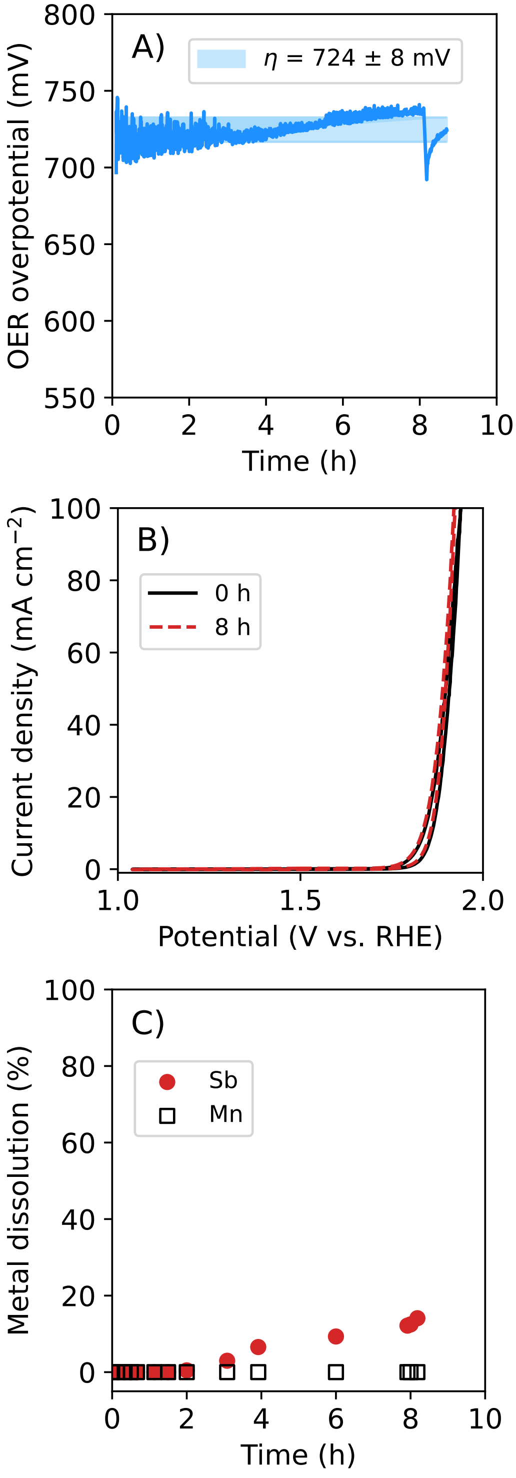

Figure S8. Electrochemical activity and stability of $Mn_{0.63}Sb_{0.37}O_x$ during OER at J = 100 mA cm^{-2} for 9 h in 1.0 M H_2SO_4(aq). Figure S8 presents a magnified view of the same data depicted in Figure 3. (A) Chronopotentiometric response. (B) Cyclic voltammograms collected both initially and after 8 h of the galvanostatic hold. (C) Corrosion products in the anolyte, as determined by ICP-MS.

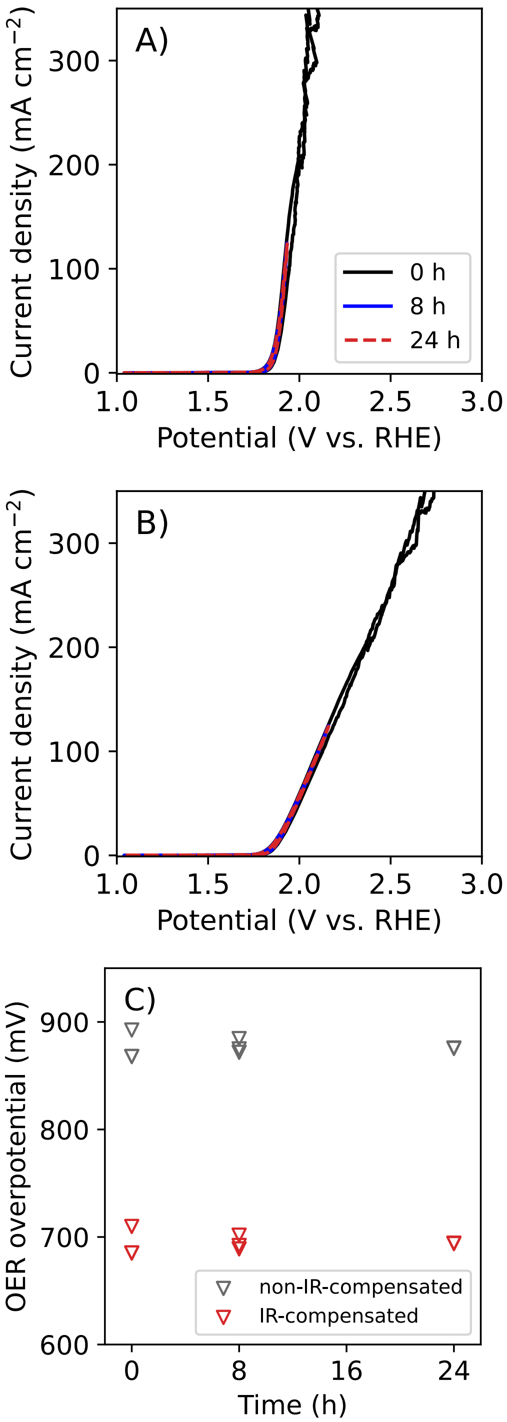

Figure S9. Cyclic voltammograms acquired with a $Mn_{0.63}Sb_{0.37}O_x$ electrode in 1.0 M H_2SO_4(aq) at three interrupt points (*t* = 0, 8, 24 h) during a 26 h galvanostatic hold at *J* = 100 mA cm^{-2} with and without IR-compensation, respectively (A) and (B). Voltammetry data at *t* = 8 h and *t* = 24 h showed that the OER overpotential at *J* = 100 mA cm^{-2} was in accord with the OER overpotential at *t* = 0 h. (C) Additional OER overpotential data at *J* = 100 mA cm^{-2} derived from voltammetry at the three interrupt points during the galvanostatic hold shown with and without IR-compensation.

t (h)	*J* (mA cm^{-2})	η (mV)	IR-compensation (mV)
0	350	819	639
0	100	709	183
8	100	688	183
24	100	694	181

Table S4. OER overpotential (η) and IR-compensation of $Mn_{0.64}Sb_{0.36}O_x$ derived from voltammetry data at J = 350 and 100 mA cm^{-2} at three interrupt points (t = 0, 8, 24 h) during a 26 h galvanostatic hold at J = 100 mA cm^{-2} in 1.0 M H_2SO_4(aq).

References

(S1) Moreno-Hernandez, I. A.; MacFarland, C. A.; Read, C. G.; Papadantonakis, K. M.; Brunschwig, B. S.; Lewis, N. S. Crystalline Nickel Manganese Antimonate as a Stable Water-Oxidation Catalyst in Aqueous 1.0 M H_2SO_4. *Energy Environ. Sci.* **2017**, *10*, 2103–2108.

(S2) Ilton, E. S.; Post, J. E.; Heaney, P. J.; Ling, F. T.; Kerisit, S. N. XPS Determination of Mn Oxidation States in Mn (Hydr)Oxides. *Appl. Surf. Sci.* **2016**, *366*, 475–485.

(S3) Biesinger, M. C.; Payne, B. P.; Grosvenor, A. P.; Lau, L. W. M.; Gerson, A. R.; Smart, R. St. C. Resolving Surface Chemical States in XPS Analysis of First Row Transition Metals, Oxides and Hydroxides: Cr, Mn, Fe, Co and Ni. *Appl. Surf. Sci.* **2011**, *257*, 2717–2730.

(S4) Militello, M. C.; Gaarenstroom, S. W. Manganese Dioxide (MnO_2) by XPS. *Surf. Sci. Spectra.* **2001**, *8*, 200–206.

(S5) Stranick, M. A. Mn_2O_3 by XPS. *Surf. Sci. Spectra.* **1999**, *6*, 39–46.

(S6) Nelson, A. J.; Reynolds, J. G.; Roos, J. W. Core-Level Satellites and Outer Core-Level Multiplet Splitting in Mn Model Compounds. *J. Vac. Sci. Technol. A.* **2000**, *18*, 1072–1076.
